# ATP-sensitive inwardly rectifying potassium channel regulation of viral infections in honey bees

**DOI:** 10.1038/s41598-017-09448-y

**Published:** 2017-08-17

**Authors:** Scott T. O’Neal, Daniel R. Swale, Troy D. Anderson

**Affiliations:** 1 Department of Entomology, Virginia Tech, Blacksburg, VA USA; 2 Department of Entomology, Louisiana State University AgCenter, Baton Rouge, LA USA; 3 Department of Entomology, University of Nebraska, Lincoln, NE USA

## Abstract

Honey bees are economically important pollinators of a wide variety of crops that have attracted the attention of both researchers and the public alike due to unusual declines in the numbers of managed colonies in some parts of the world. Viral infections are thought to be a significant factor contributing to these declines, but viruses have proven a challenging pathogen to study in a bee model and interactions between viruses and the bee antiviral immune response remain poorly understood. In the work described here, we have demonstrated the use of flock house virus (FHV) as a model system for virus infection in bees and revealed an important role for the regulation of the bee antiviral immune response by ATP-sensitive inwardly rectifying potassium (K_ATP_) channels. We have shown that treatment with the K_ATP_ channel agonist pinacidil increases survival of bees while decreasing viral replication following infection with FHV, whereas treatment with the K_ATP_ channel antagonist tolbutamide decreases survival and increases viral replication. Our results suggest that K_ATP_ channels provide a significant link between cellular metabolism and the antiviral immune response in bees.

## Introduction

Honey bees (*Apis mellifera*) play an economically vital role in global agriculture as pollinators of a wide variety of crops, in addition to being valued for the honey and other natural products that they provide. Declines in the numbers of both managed and wild pollinators have served to increase public awareness of bee health issues and prompted researchers to increase efforts to understand the factors driving these declines. While there exist a variety of factors that negatively impact the health and survival of both managed and wild bee populations, there is a growing consensus that the prevalence of parasites and pathogens, especially viruses, are among the most significant threats to pollinator health^1–3^. Viral infections have been found to be closely associated with weakened or dying bee colonies, as well as with colonies believed to be affected by Colony Collapse Disorder (CCD)^4–7^. The viruses that most commonly infect bees are nonenveloped, positive sense, single-stranded RNA viruses belonging to either the family *Dicistroviridae* or *Iflaviridae* in the order *Picornavirales* ^8^. Bee colonies are commonly infected with multiple viruses concurrently^9–11^, and those infections may range from asymptomatic to producing overt deformities, paralysis, and even death. There exist multiple routes of transmission for bee viruses^12^, the most damaging being the ectoparasitic mite *Varroa destructor*, which can suppress overall immune responsiveness and cause seemingly harmless chronic infections to become acute and devastating^13, 14^. Evidence suggests that this complex interplay between host, pathogen, and parasite significantly alters the critical balance that exists between viral replication strategies and the host immune defenses and is thought to be a significant factor driving colony loss^5, 13^.

One of the primary invertebrate antiviral defense mechanisms is an evolutionarily-conserved, post-transcriptional gene silencing mechanism known as RNA interference (RNAi), which recognizes the presence of double-stranded RNA to initiate targeted RNA degradation^15, 16^. Insects also employ the Janus kinase and Signal Transducer and Activator of Transcription (Jak/STAT), Toll, and Immune deficiency (Imd) innate immune response pathways in response to viral infections, though the importance of these seems to vary^17–19^. The bee genome has been demonstrated to encode the major proteins associated with these immune pathways^20^, and bees have been shown to utilize an antiviral RNAi response^21–24^. Interestingly, however, bees have been found to possess only about one third as many genes associated with insect immunity as fruit flies and mosquitoes^25^, possibly suggesting a reduced reliance on the immune response of the individual. This discrepancy may be explained by the effectiveness of social immune barriers to infection that function at the colony level or it is possible that bees also rely on homeostatic mechanisms to control damage at the level of the cell or tissue to simply tolerate infection^26^. Studies using the fruit fly *Drosophila melanogaster*, for example, have shown that Jak/STAT deficient flies are still able to resist infection with high titers of virus, despite a reduced transcriptional response to infection and an increased viral load^27^. Conversely, RNAi deficient flies have been observed to experience rapid mortality in response to viral infections, despite experiencing viral loads no greater than those observed in wild type controls^28^. Similar findings have demonstrated the importance of the heart in maintaining homeostasis during the innate immune response of both mammals and insects during a viral infection and suggest that ATP-sensitive inwardly rectifying potassium (K_ATP_) channels play an evolutionarily conserved role in mediating this interaction between the host and the virus^29^.

K_ATP_ channels belong to the family of inwardly rectifying potassium (K_ir_) channels, which are a class of potassium (K^+^) selective ion channels that conduct larger inward currents at membrane potentials negative to the equilibrium potential of K^+^ than outward currents at potentials positive to it under physiological conditions^30^. K_ir_ channels mediate K^+^ transport across the membrane and are responsible for stabilizing the resting membrane potential of the cell and for regulating action potential duration in electrically excitable cells such as cardiac muscle^30^. K_ATP_ channels are heteromeric octamers composed of four pore-forming K_ir_ channel subunits and four regulatory sulfonylurea receptor (SUR) subunits^31^ that are regulated by the relative level of ATP present in the cytosol and consequently provide an important link between the metabolic state of the cell and its membrane potential^30^. In mammals, roles for K_ATP_ channels have been described in a variety of different tissues, including heart, skeletal muscle, vascular smooth muscle, pancreas, and brain^32^. While much of the research investigating K_ATP_ channels focuses on their role in the sarcolemma and the cytoplasmic membrane, it is worth noting that K_ATP_ channels have also been identified in the mitochondrial membrane^33^. Evidence suggests that mammalian K_ATP_ channels facilitate cardiovascular tolerance to endotoxic shock by linking vasoreactivity to metabolic demand, allowing coronary smooth muscle cells to adapt to the systemic stress caused by the innate immune response to infections^34^. Relative to mammals, considerably less is known about the physiological role of insect K_ir_ channels and K_ATP_ channels. The majority of research focused on insect K_ir_ channels has been conducted using fruit flies^35^ and mosquitoes^36^, though a recent study has added bedbugs to the list^37^. Much of this research has focused on the role of K_ir_ channels in Malpighian tubule function and their potential for exploitation as a novel insecticide target in *Aedes* and *Anopheles* mosquitoes^38–40^. Research related to the insect innate immune response has found that K_ATP_ channels appear to play a role in mediating the survival of *Drosophila* during viral infections similar to that observed in mammals^29^. This finding has led to the further observation that insect K_ATP_ channels have a function in modulating antiviral RNAi, presumably by facilitating tissue-specific regulation of innate immune response mechanisms by the cellular environment of the heart^41^.

At this time, very little is known about the role of K_ir_ channels in the honey bee or any other hymenopteran. Recently published findings have shown that K_ATP_ channel modulators can significantly alter heart rate in the honey bee^42^, but there is no information available about the relationship between K_ATP_ channels and the antiviral immune response of bees. Unfortunately, investigations of bee antiviral immune responses must contend with several major obstacles. One of those obstacles is that bee colonies are often covertly infected with one or more viruses at any given time, posing a challenge for researchers focused on the outcome of infection with a single virus. Another is that infectious clones for honey bee viruses have not been developed, despite the fact that numerous bee viruses have been sequenced^43–49^. Some research has been conducted using semi-purified virus preparations^8^, but complete removal of contaminating viruses is often impossible, making it difficult to accurately characterize infection dynamics. Recent work has proposed and tested Sindbis virus expressing enhanced green fluorescent protein as an experimental model of honey bee virus infection, using it to show that nonspecific double-stranded RNA triggers an antiviral response that controls viral infection in bees^50^. In this study, we demonstrate the use of a different virus, flock house virus (FHV), as another experimental model for the study of viral infections in bees. FHV belongs to the family *Nodaviridae*, whose members are nonenveloped, icosahedral viruses with genomes composed of two single-stranded, positive sense RNAs^51^. FHV has been well characterized structurally and genetically and has served as a model system for the study of host-virus interactions in a variety of arthropod species^52^. Here we report that FHV infects adult bees, causing rapid onset of mortality and accumulation of viral RNA. Furthermore, infection-mediated mortality can be altered by pre-exposure to K_ATP_ channel modulators. Pharmacological activation of K_ATP_ channels reduced the onset of mortality and the level of viral RNA production following FHV infection, whereas inhibition of K_ATP_ channels increased both the rate of mortality and viral replication.

## Results

### FHV can infect honey bees

**Figure 1:**
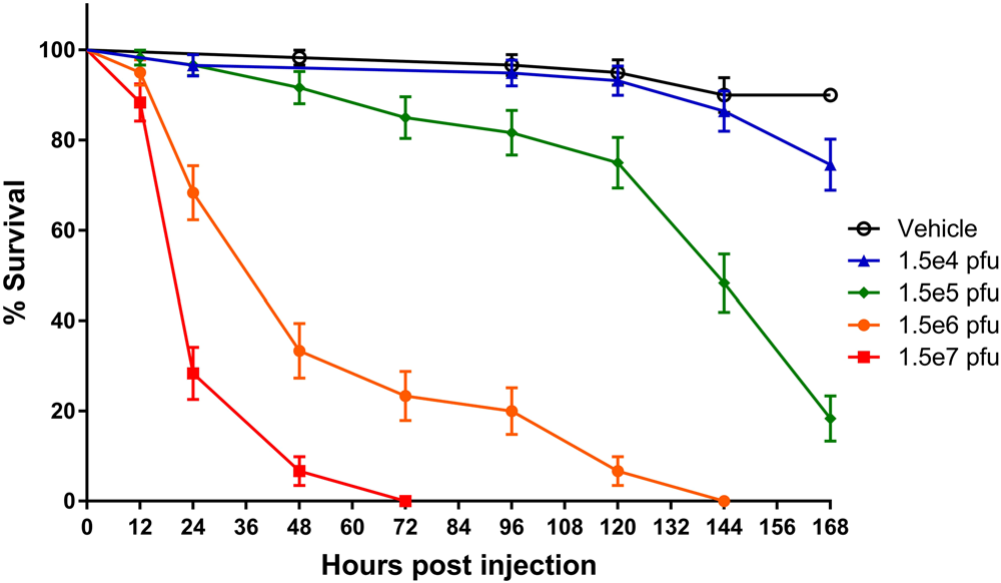
Honey bee survival following infection with increasing titers of FHV. Data presented as Kaplan-Meier survival curves with points representing mean values ± standard error for 150 bees (6 replicate groups of 25 adult bees). Groups receiving 1.5 × 10^5^ pfu of FHV/bee or more experienced significantly higher mortality than vehicle control group (Kaplan-Meier log-rank test; *P* < 0.0001). Data analyzed using GraphPad Prism 7 software.

**Figure 2:**
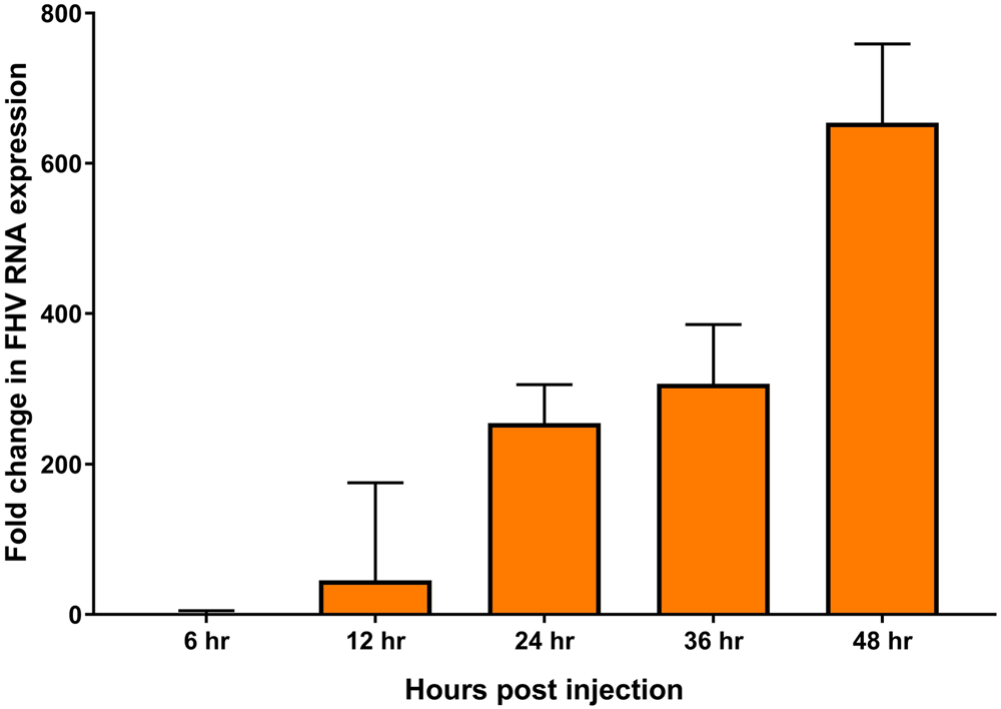
Fold change in FHV RNA expression over time following infection with 1.5 × 10^6^ pfu of FHV/bee. Expression of FHV RNA1 was measured and presented as the mean fold change (RQ ± RQmax/RQmin) relative to the amount of virus present at the time of infection. Time points represent 3 technical replicates of a pooled sample of 6 bees.

Intrathoracic injection of gradient-purified FHV into honey bees resulted in high mortality. The time to death was concentration-dependent, with increasing viral titers resulting in more rapid mortality (Fig. 1). Bees injected with the highest titer of virus, receiving approximately 1.5 × 10^7^ plaque-forming units (pfu) of FHV/bee, experienced 70% mortality within the first 24 h and 100% mortality by 72 h post injection. Bees injected with 1.5 × 10^6^ pfu of FHV/bee experienced 30% mortality within the first 24 h and 100% mortality was not observed until 144 h post injection. Bees injected with 1.5 × 10^5^ pfu of FHV/bee or 1.5 × 10^4^ pfu of FHV/bee were indistinguishable from vehicle controls at 24 h post injection and 100% mortality was not observed within the time period monitored during this study. Log-rank tests of the Kaplan-Meier survival curves indicated a significant difference in survival between bees challenged with 1.5 × 10^5^ pfu of FHV/bee or more and bees challenged with vehicle (Kaplan-Meier log-rank test; *P* < 0.0001). Increasing amounts of FHV RNA1 were detected in infected bees using quantitative real-time PCR (qPCR) (Fig. 2), indicating FHV RNA replication. Furthermore, accumulation of FHV RNA was detected in naïve bees that were injected with virus particles isolated from FHV-infected bees, demonstrating infectivity of the virus (Figure S1). These data demonstrate that FHV is capable of productively infecting bees and suggest that FHV may serve as a model of virus infection in bees.

### K_ATP_ channel modulators affect the outcome of FHV infection in bees

**Figure 3:**
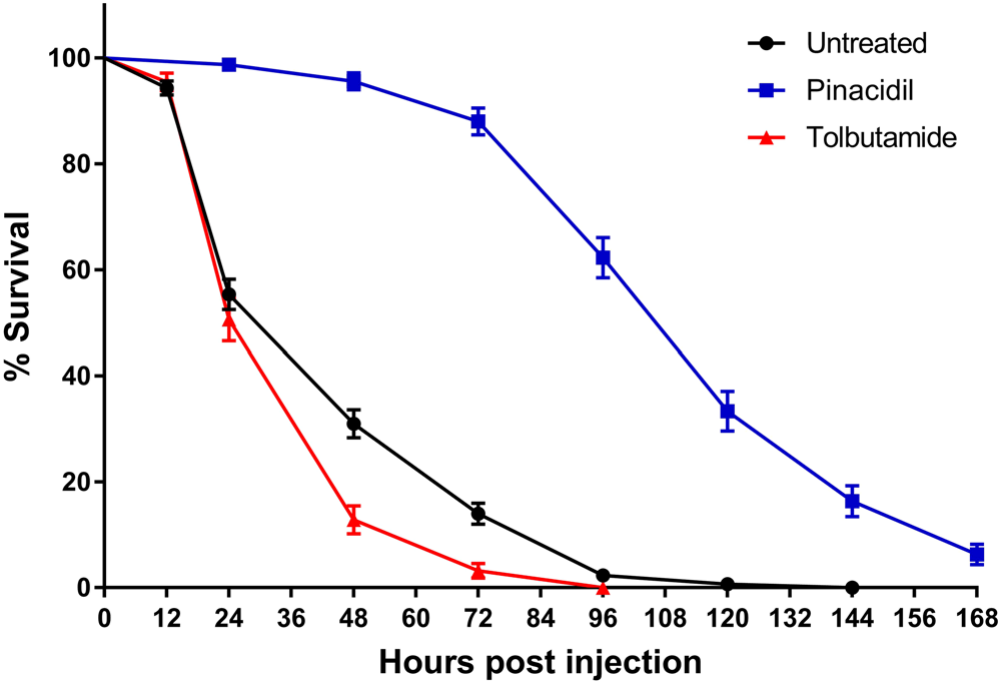
Honey bee survival following pinacidil or tolbutamide treatment and infection with 1.5 × 10^6^ pfu of FHV/bee. Data presented as Kaplan-Meier survival curves with points representing mean values ± standard error for 150 bees (6 replicate groups of 25 adult bees). Bees receiving pinacidil experienced significantly lower mortality than untreated bees (Kaplan-Meier log-rank test; *P* < 0.0001). Bees receiving tolbutamide experienced significantly higher mortality than untreated bees (Kaplan-Meier log-rank test; *P* < 0.001). Data analyzed using GraphPad Prism 7 software.

Treatment with the K_ATP_ channel agonist pinacidil increased survival of honey bees following infection with 1.5 × 10^6^ pfu of FHV/bee, whereas treatment with the K_ATP_ channel antagonist tolbutamide decreased survival, relative to a untreated control (Fig. 3). Untreated bees experienced 65% mortality within the first 48 h and 100% mortality by 144 h post injection. Pinacidil-treated bees experienced 5% mortality within the first 48 h and 100% mortality was not observed within the time period monitored during this study. Tolbutamide-treated bees experienced 87% mortality within the first 48 h and 100% mortality by 96 h post injection. Log-rank tests of the Kaplan-Meier survival curves indicated a significant difference in survival between untreated bees and both bees treated with pinacidil (Kaplan-Meier log-rank test; *P* < 0.0001) and bees treated with tolbutamide (Kaplan-Meier log-rank test; *P* < 0.001). When infected with a higher titer of virus (1.5 × 10^7^ pfu of FHV/bee), survival in pinacidil-treated bees was somewhat less pronounced, but was still significantly increased relative to untreated bees (Kaplan-Meier log-rank test; *P* < 0.0001) (Figure S2). When infected with a lower titer of virus (1.5 × 10^5^ pfu of FHV/bee), mortality in tolbutamide-treated bees became more pronounced and survival was significantly decreased relative to untreated bees (Kaplan-Meier log-rank test; *P* < 0.0001) (Figure S3).

**Figure 4:**
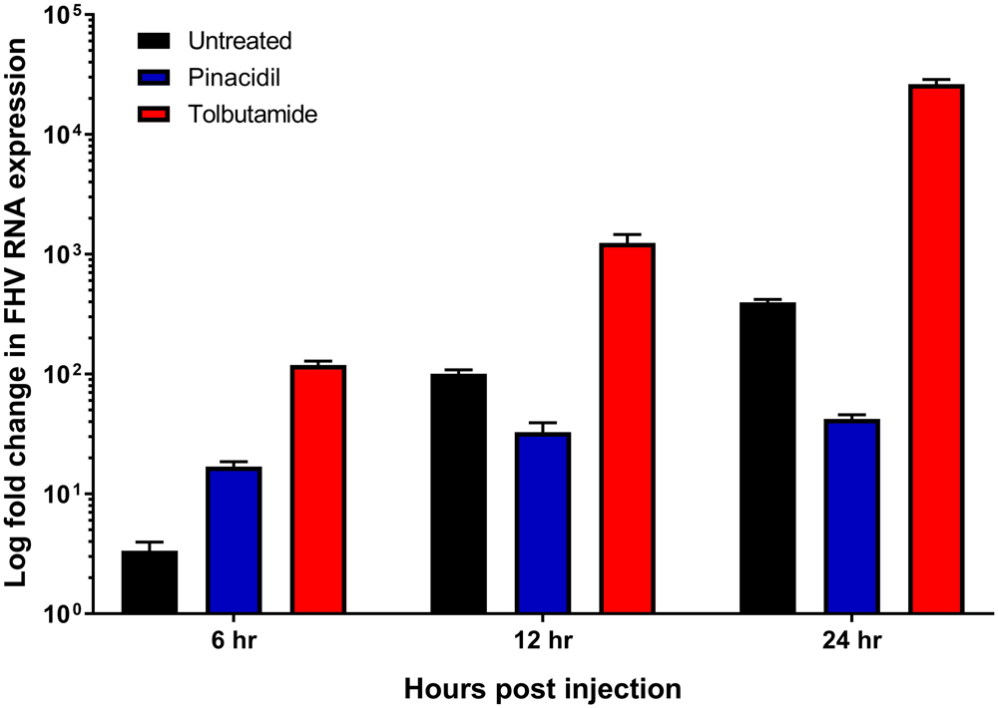
Log_10_ fold change in FHV RNA expression over time following pinacidil or tolbutamide treatment and infection with 1.5 × 10^6^ pfu of FHV/bee. Expression of FHV RNA1 was measured and presented as the log_10_ mean fold change (RQ ± RQmax/RQmin) relative to the amount of virus present at the time of infection. Time points represent 4 technical replicates of a pooled sample of 6 bees.

In order to confirm that K_ATP_ channel modulators are affecting viral replication, relative changes in the amount of FHV RNA1 were assessed using qPCR following treatment with pinacidil or tolbutamide (Fig. 4). Pinacidil treatment led to decreased accumulation of viral RNAs within 24 h post injection, with a one-log lower fold change in expression when compared to untreated controls. Tolbutamide treatment led to increased accumulation of viral RNAs within the first 24 h post injection, with a two-log higher fold change in expression when compared to untreated controls. A pathogen screen of the source colony detected the presence of deformed wing virus (DWV), a common honey bee virus that is widely present as a low level, covert infection in managed colonies^8^. In order to determine whether or not infection with FHV had an effect on existing DWV infections, the presence of DWV and changes in its expression were also assessed using qPCR in the same samples that were tested for FHV expression following treatment with pinacidil or tolbutamide (Figure S4). In general, for all three treatment groups at each time point observed, expression of DWV RNA was lower than what was observed at the time of infection with FHV, with the exception of the pinacidil-treated group at 12 h, which showed a slightly higher level of expression. It should be noted that only limited conclusions can be drawn from this data, as there is no way to control for the initial level of DWV infection in the bees sampled. These results, however, do not show any evidence of increased DWV virulence as a result of FHV infection, suggesting that the mortality observed in infected bees is the result of FHV infection.

## Discussion

Our findings demonstrate the use of FHV as an experimental model for the study of viral infections in honey bees and reveal an evolutionarily conserved relationship between K_ATP_ channels and the antiviral immune response of bees. Despite the economic and agricultural importance of this domesticated pollinator species and the growing awareness of the role of viral pathogens in bee declines, the mechanisms involved in the antiviral immune response of bees remain poorly characterized. This represents a critical gap in our knowledge of the complex relationship between host and pathogen that must be filled if we are to better understand how viruses and immune deficiencies affect bee health and to develop more effective strategies to mitigate colony loss.

The establishment of FHV as a model system for virus infection in bees helps to avoid some of the major challenges facing investigations of the bee antiviral immune response, which include the lack of infectious clones of bee viruses and the prevalence of covert viral infections already present in many colonies. The use of FHV as a model virus should ensure that the bees used in an experiment will not be previously infected, as FHV is not a pathogen normally found in bees. Despite not being a naturally occurring bee virus, FHV bears much similarity to bee viruses in general, as it is a nonenveloped, positive sense, single-stranded RNA virus of insect origin^53^. FHV is a well-studied and extensively characterized virus that has an established history of use in studies investigating antiviral immunity in other insect species^52^. FHV can be readily produced and purified in the lab, allowing investigators to precisely deliver a known quantity of a single virus during infection, which is not necessarily the case when using a semi-purified inoculum of a bee virus. Precision is further enhanced by infection via intrathoracic injection, rather than infection via an oral route, which ensures that the same amount of infectious material is administered to each test subject. FHV also offers the opportunity to observe how the bee antiviral immune response copes with a novel viral challenge, rather than a virus that may be specifically adapted to persistently infect a bee host, thus simulating the challenges that would be faced by the introduction of either a novel virus or an established virus that has gained increased virulence. FHV possesses a known and well-studied suppressor of RNAi, the B2 protein, which inhibits processing of viral RNA by Dicer nuclease, rather than suppression of immune response genes or action through some unknown mechanism, as could be the case with other viruses that have not been as thoroughly characterized. Although it would be preferable in many cases to study the effects of a virus that has co-evolved with bees as a host, there are distinct advantages at this time to the use of FHV as an experimental model for the study of viral infections in bees.

Using FHV as a model system in bees, we have shown that K_ATP_ channels appear to have an important function in the tolerance of bees to viral infection. In *Drosophila*, this function has been shown to rely on modulation of RNAi^41^, which serves as one of the primary invertebrate antiviral defense pathways^16^. What this finding describes is a similar role for ion channels in the regulation of the honey bee antiviral immune response, in particular, ion channels that are sensitive to changes in the cellular environment. In mammals, K_ATP_ channels are understood to have an important role in the maintenance of cardiac cellular homeostasis and functional K_ATP_ channels have been demonstrated as a requirement for successful adaptation to stress^54^. Due to their presence in metabolically active tissues and integration with both cellular and systemic metabolic processes, K_ATP_ channels have been described as a unifying molecular coordinator of metabolic well-being under stress^55^. Evidence suggests that this evolutionarily conserved function is present in insects as well^29, 41^, leading us to hypothesize that K_ATP_ channels provide an important link between stress and reduced immunocompetence in bees. Exposure to chronic stress is understood to gradually weaken the immune response and reduce metabolic activity of an organism until it is no longer capable of survival^56^. Bees are no exception, as physiological stress can have a range of detrimental consequences for bee health and survival^57^. A variety of studies have demonstrated harmful synergistic interactions between simultaneous exposure to pesticides, dietary toxins, and pathogens in bees, but the mechanisms that explain these interactions remain unknown^58–61^. K_ATP_ channels effectively serve as a sensor or feedback mechanism that responds to changes in the metabolic state of a cell, as one of their defining characteristics is that they are gated by the relative levels of intracellular nucleotides, primarily ATP and ADP^30^. Exposure to a wide range of environmental stressors can effectively depress or downregulate the metabolic activity of an organism, which includes ATP production and turnover^62^. Consequently, K_ATP_ channels are in a unique position to link stress-induced changes in bee metabolism to the antiviral immune response.

In summary, we have demonstrated the effectiveness of FHV as a model system for virus infection in honey bees, but more importantly, we have revealed an important role for ion channel regulation of the honey bee antiviral immune response. We have shown that treatment with the K_ATP_ channel agonist pinacidil increases survival of honey bees while decreasing viral replication following infection with FHV, whereas treatment with the K_ATP_ channel antagonist tolbutamide decreases survival and increases viral replication. Based on our findings and what is known about the role of the evolutionarily conserved K_ATP_ channel in other systems, we propose an important role for this ion channel in connecting the antiviral immune response of bees to changes in the cellular environment induced by environmental stressors. Additional work is necessary to confirm this hypothesized role of K_ATP_ channels and to expose the mechanisms of their relationship to stress and the immune response, but this represents a promising area for future research designed to enhance our understanding of honey bee immune responses and the factors that negatively impact pollinator health.

## Methods

### Subjects

European honey bees (*Apis mellifera*) were used for all experiments. Bee colonies were maintained as previously described^63^ according to standard beekeeping practices using commercial hives that were housed in an apiary located at the Virginia Tech Price’s Fork Research Facility (Blacksburg, VA). For all laboratory experiments, frames of emerging worker brood were removed from the hive and housed in an incubator at 32 °C with a relative humidity of 50–80%. Newly emerged bees were collected from these frames over the course of 48 h and sorted into cages in groups of approximately 25 per cage. All cages were also provided with ¼ portions of a queen mandibular pheromone (QMP)-impregnated strip (Mann Lake Ltd.) to reduce stress by simulating the presence of an egg-laying queen. Bees remained housed in incubators under these conditions for the duration of all experiments.

### Drug treatment

Stock solutions of tolbutamide and pinacidil (Sigma Aldrich) were initially prepared in dimethyl sulfoxide (DMSO). Cages of newly emerged bees were provided *ad libitum* access to a 50% solution of sucrose in water. Test groups received sucrose solution supplemented with 1% DMSO and either tolbutamide or pinacidil (2 mM final concentration), while untreated control groups received sucrose solution supplemented only with 1% DMSO. Preliminary testing demonstrated no significant effects of DMSO on either bee mortality or viral replication. For all experiments, bees were provided access to the same sucrose solution for the duration of the test. For all experiments lasting more than 48 h, bees were also provided *ad libitum* access to honey starting at 72 h.

### Infections with purified virus

All experiments involving viral infections utilized FHV, kindly provided by Dr. Anette Schneemann (The Scripps Research Institute, La Jolla, California), that was purified as previously described^64^. Viral stocks were prepared in 10 mM Tris-HCl, pH 7.5. Infections were performed by injection (Nanoject II apparatus; Drummond Scientific) of 50.6 nl of a viral suspension into the thorax of each bee. Injection of the same volume of 10 mM Tris-HCl, pH 7.5, was used as an injection control. For all experiments involving drug treatments, bees were injected following 24 h of exposure to drug-supplemented sucrose solution as the only source of food and water.

### Survival

Preliminary testing compared survival of bees following vehicle control injections and sham injections, in which the bee thorax was punctured by the needle without delivery of fluid, and found that neither group experienced significantly greater mortality than uninjected bees over a 7 d period. For all survival experiments, six replicates of approximately 25 bees were used for each treatment group. Survival was observed 12 h following injection and then every 24 h for 7 d. In order to assess the effect of infection with increasing titers of FHV on bee mortality, groups of bees were injected with approximately 1.5 × 10^7^ pfu of FHV/bee (50.6 nl of 3 × 10^8^ pfu/µl viral suspension), 1.5 × 10^6^ pfu of FHV/bee, 1.5 × 10^5^ pfu of FHV/bee, or 1.5 × 10^4^ pfu of FHV/bee and survival was compared to injection controls. In order to assess the effect of tolbutamide and pinacidil treatment on survival following infection with FHV, bees were treated with either drug-supplemented or unsupplemented sucrose solution and then injected with either virus or a vehicle control. Pinacidil was tested against 1.5 × 10^6^ pfu of FHV/bee and 1.5 × 10^7^ pfu of FHV/bee. Tolbutamide was tested against a viral challenge of 1.5 × 10^6^ pfu of FHV/bee and 1.5 × 10^5^ pfu of FHV/bee.

### Viral RNA expression

In order to assess the level of viral replication in bees, a group of otherwise untreated bees was injected with 1.5 × 10^6^ pfu of FHV/bee. In order to assess the effect of tolbutamide and pinacidil treatment on FHV RNA expression following infection, groups of bees were fed either unsupplemented or drug-supplemented sucrose solution, as described for survival studies, and then injected with 1.5 × 10^6^ pfu of FHV/bee. For all treatments, 6 bees were collected at each time point, dissected, and then frozen together in liquid nitrogen for pooled analysis of RNA expression. Frozen samples were maintained at −80 °C until RNA isolation was performed. The head and abdomen were removed from each insect, as PCR inhibitors have been noted in the eye^65^ and preliminary experiments found that inclusion of the gut also inhibited PCR. The first time point for each treatment (0 h) was collected immediately following injection to be used as a calibrator, or reference sample, and subsequent time points were collected at 6, 12, and 24 h following infection.

### Infections with virus isolate

In order to demonstrate infectivity, groups of otherwise untreated bees were injected with approximately 1.5 × 10^7^ pfu of FHV/bee and after 24 h, surviving bees were homogenized in groups of approximately 10 individuals in 10 mM Tris-HCl, pH 7.5. Virus particles were collected from the supernatant and concentrated using a polyethylene glycol (PEG) virus precipitation kit (Biovision) according to manufacturer instructions. Groups of bees were then injected with virus isolate as described for infections with purified virus. At 0, 24, and 48 h post-injection, 6 bees were collected for pooled analysis of RNA expression, as described for viral RNA expression.

### Primers and probes

The following primers and probe were designed for the detection of FHV RNA1: forward 5′-GGACCGAAGTGCGGTGATG-3′, reverse 5′-CAGTTTTGCGGGTGGGGGG-3′, and probe FAM-5′-TGCCGCAATGAAGGATGTCT-3′-TAMRA. Primers and probe used for the detection of β-actin have been previously described^66^: forward 5′-AGGAATGGAAGCTTGCGGTA-3′, reverse 5′-AATTTTCATGGTGGATGGTGC-3′, and probe FAM-5′-ATGCCAACACTGTCCTTTCTGGAGGTA-3′-TAMRA. Primers and probe used for the detection of DWV RNA have also been previously described^66^: forward 5′-ATCAGCGCTTAGTGGAGGAA-3′, reverse 5′-TCGACAATTTTCGGACATCA-3′, and probe FAM-5′-CGCATGAACAAGTTCGGCGTT-3′-6-TAMRA.

### Quantitative PCR

Total RNA was isolated from each sample using TRI Reagent RT (Molecular Research Center, Inc.) and then 1 µg was reverse transcribed using a high capacity cDNA reverse transcription kit (Applied Biosystems). The thermal cycling parameters used for reverse transcription were 25 °C for 10 min, 37 °C for 120 min, and then 85 °C for 5 min. Gene expression was determined using qPCR on a StepOne Real-Time PCR System (Applied Biosystems). Each PCR reaction (20 µl) contained TaqMan Fast Advanced Master Mix (Applied Biosystems), 100 ng cDNA template, 1 µM forward and reverse primers, and 250 nM TaqMan probe. The thermal cycling parameters used for qPCR were 95 °C for 20 sec for enzyme activation followed by 40 cycles of denaturation at 95 °C for 1 s and annealing/extension at 60 °C for 20 s.

The comparative C_T_ (ΔΔC_T_) method, which determines the quantity of target in a sample relative to the quantity of target in a reference sample^67, 68^, was used to assess the change in FHV RNA1 expression at each time point relative to the 0 hr sample. Measurements of target gene expression were normalized using *Apis mellifera* β-actin as the endogenous control. Three technical replicates were analyzed for untreated bees injected with 1.5 × 10^6^ pfu of FHV/bee and four technical replicates were analyzed for all other samples. Serial dilutions of different inputs of target and reference genes were performed to verify that efficiencies of target and reference are approximately equal. Viral RNA expression is reported as the mean fold change, measured in relative quantity units (RQ) ± RQmax/RQmin, relative to the calibrator sample for each treatment. Because the relative quantitation is exponentially related to the threshold value (C_T_), calculation of the statistical error results in an asymmetric distribution of the upper and lower limits, precluding direct statistical comparisons between expression levels in different treatment groups. Data analysis and calculation of RQ, RQmax, and RQmin were performed using StepOne Software v2.3 (Applied Biosystems).

### Statistical Analysis

All data analysis and graph construction was performed using GraphPad Prism 7 (GraphPad Software, Inc., La Jolla, CA). Survival experiment results are reported as Kaplan-Meier survival curves, displaying mean values ± standard error, with significant differences between the survival curves determined by the log-rank (Mantel-Cox) test.

## Electronic supplementary material

Supplemental Figures
