# Supplementary material for: ATP-sensitive inwardly rectifying potassium channel regulation of viral infections in honey bees
Source: Sci Rep. 2017 Aug 17;7:8668. doi: 10.1038/s41598-017-09448-y (PMC5561242; doi:10.1038/s41598-017-09448-y)
Supplement: Supplementary file 1 — Supplemental Figures [file 41598_2017_9448_MOESM1_ESM.pdf]

ATP-sensitive inwardly rectifying potassium channel regulation of viral infections in honey bees

Scott T. O'Neal[1*], Daniel R. Swale[2], Troy D. Anderson[3*]

1 Department of Entomology, Virginia Tech, Blacksburg, VA, USA

2 Department of Entomology, Louisiana State University AgCenter, Baton Rouge, LA, USA

3 Department of Entomology, University of Nebraska, Lincoln, NE, USA

***** Corresponding authors: S.T.O. (onealst@vt.edu) and T.D.A. (tanderson44@unl.edu)

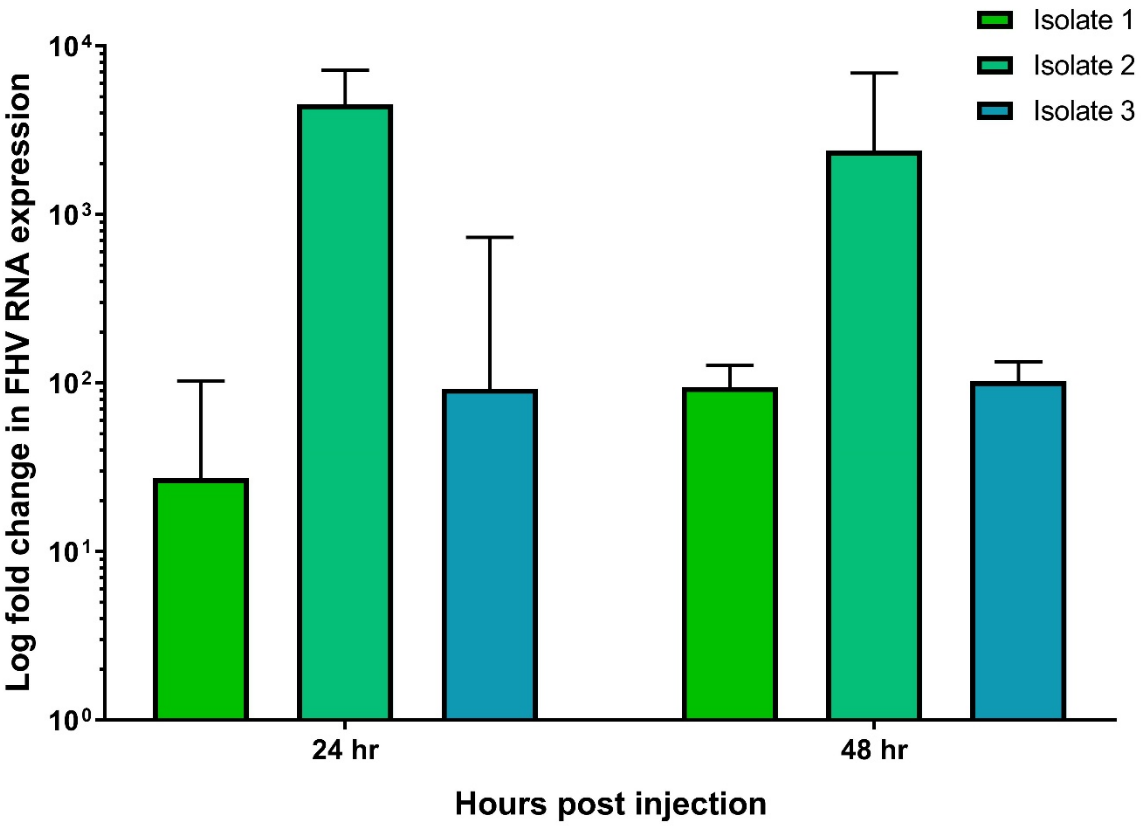

Figure S1. Log$_{10}$ fold change in FHV RNA expression over time following infection with virus particles isolated from FHV-infected bees. Expression of FHV RNA1 was measured and presented as the log$_{10}$ mean fold change (RQ ± RQmax/RQmin) relative to the amount of virus present at the time of infection. Time points represent 3 or 4 technical replicates of a pooled sample of 6 bees.

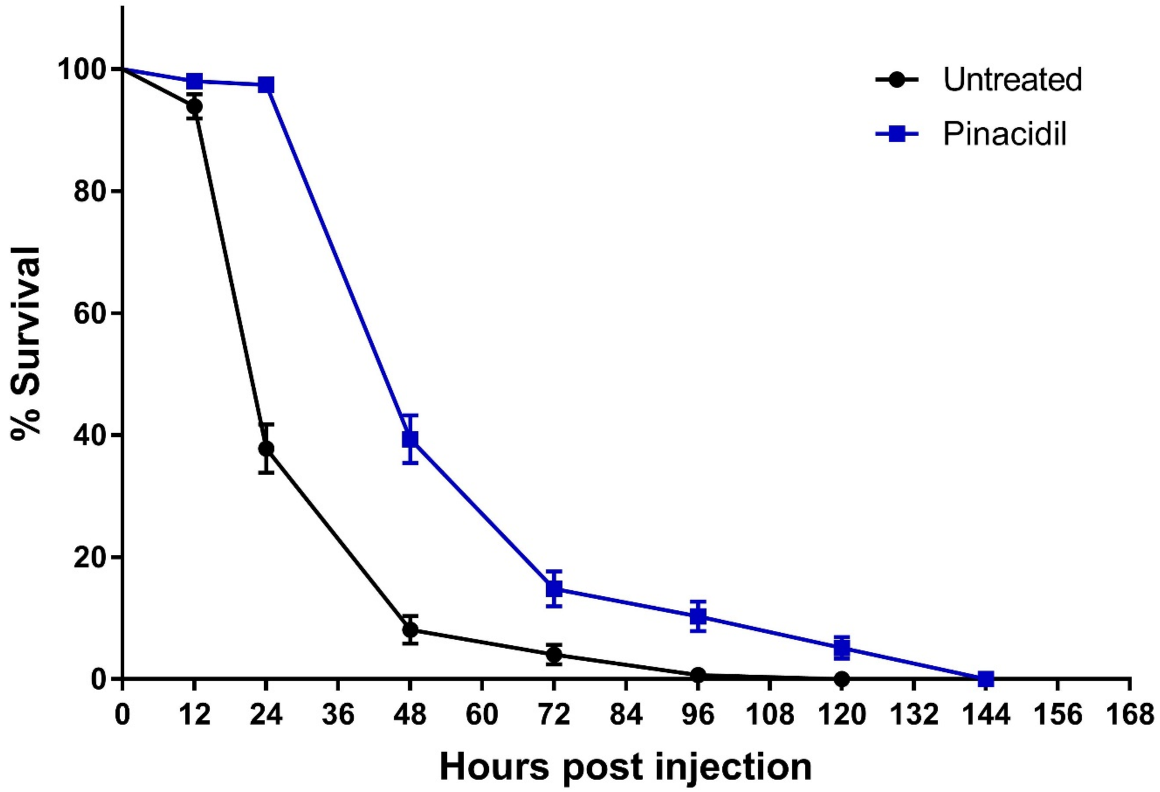

Figure S2. Honey bee survival following pinacidil treatment and subsequent infection with 1.5 x 10^7 pfu of FHV/bee. Data presented as Kaplan-Meier survival curves with points representing mean values ± standard error for 150 bees (6 replicate groups of 25 adult bees). Bees receiving pinacidil experienced significantly lower mortality than untreated bees (Kaplan-Meier log-rank test; $P < 0.0001$). Data analyzed using GraphPad Prism 7 software.

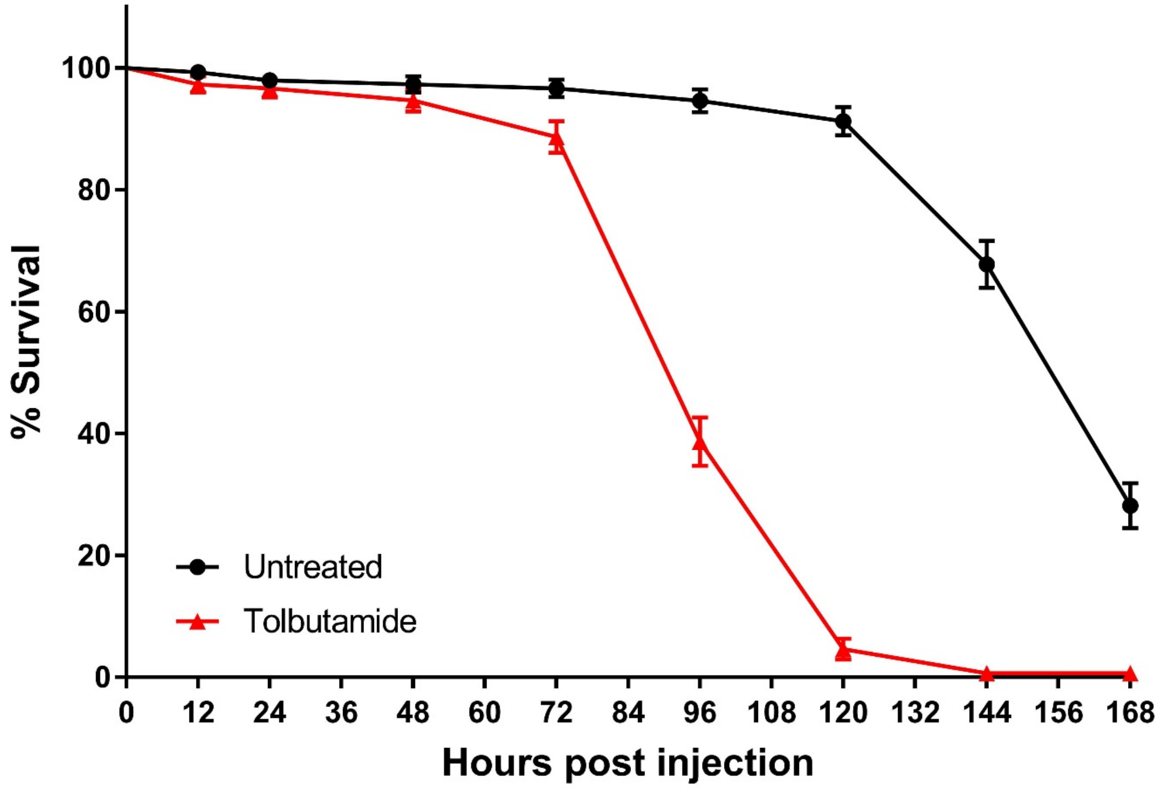

Figure S3. Honey bee survival following tolbutamide treatment and subsequent infection with 1.5 x 10^5 pfu of FHV/bee. Data presented as Kaplan-Meier survival curves with points representing mean values ± standard error for 150 bees (6 replicate groups of 25 adult bees). Bees receiving tolbutamide experienced significantly higher mortality than untreated bees (Kaplan-Meier log-rank test; $P < 0.0001$). Data analyzed using GraphPad Prism 7 software.

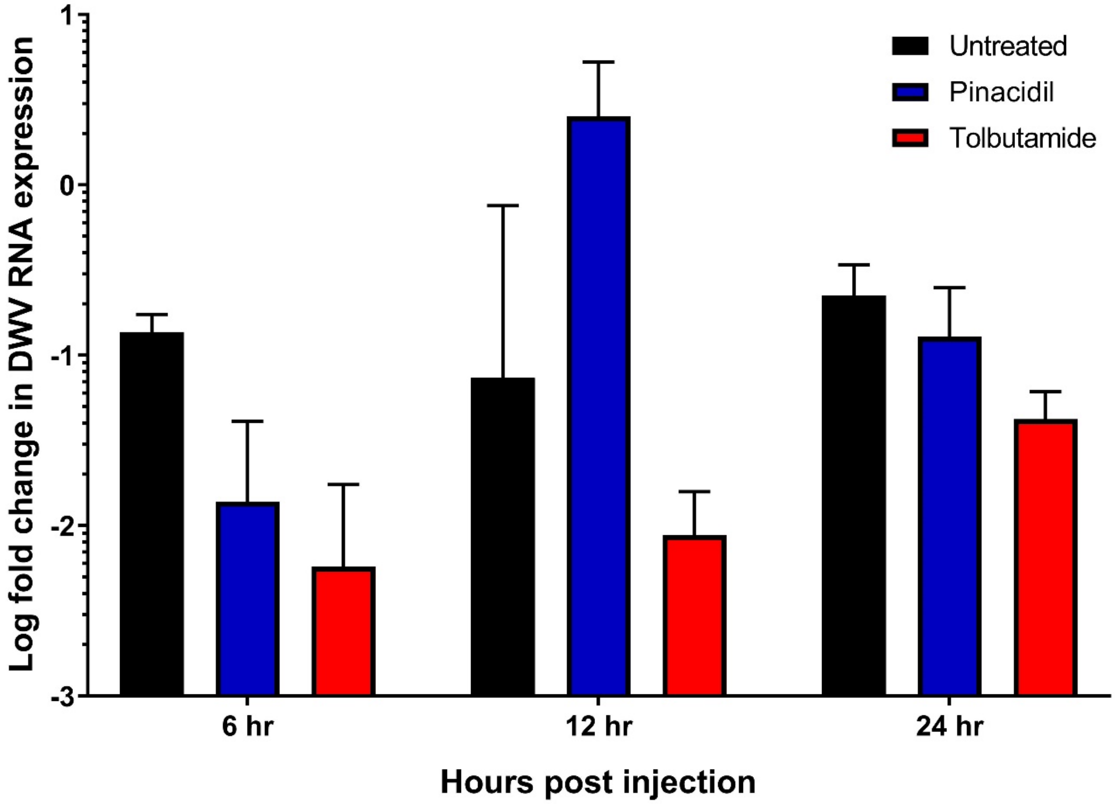

Figure S4. Log$_{10}$ fold change in DWV RNA expression over time following pinacidil or tolbutamide treatment and infection with 1.5 x 10^6 pfu of FHV/bee. Expression of DWV RNA was measured and presented as the log$_{10}$ mean fold change (RQ ± RQmax/RQmin) relative to the amount of virus present at the time of infection. Time points represent 3 technical replicates of a pooled sample of 6 bees.
